# Vertical structure of subsurface marine heatwaves in a shallow nearshore upwelling system

**DOI:** 10.1038/s41598-025-90565-4

**Published:** 2025-02-21

**Authors:** Gavin Plume, Ryan K. Walter, Isabelle Cobb, Michael Dalsin, Piero L. F. Mazzini, Nathan P. Shunk, Ian Robbins, Thomas P. Connolly

**Affiliations:** 1 Physics Department, California Polytechnic State University, San Luis Obispo, CA USA; 2 Virigina Institute of Marine Science, William and Mary, Gloucester Point, VA USA; 3 Moss Landing Marine Laboratories, San José State University, Moss Landing, CA USA

**Keywords:** Physical oceanography, Physical oceanography

## Abstract

Marine heatwaves (MHWs) are increasing in frequency and intensity globally and are among the greatest threats to marine ecosystems. However, limited studies have characterized subsurface MHWs, particularly in shallow waters. We utilized nearly two decades of full water-column (~ 10 m) observations from a unique automated profiler in central California to characterize, for the first time, the vertical structure of MHWs in a shallow nearshore upwelling system. We found MHWs have similar average durations and intensities across all depths, but there were ~ 17% more bottom MHW days than surface MHW days. Nearly one third of bottom MHWs occurred independently of surface MHWs, indicating that satellites miss a significant fraction of events. MHWs showed distinct seasonality with more frequent and intense events during the fall/winter when weak stratification allowed for MHWs to occupy a larger portion of the water column and persist longer. During summer, strong stratification limited the vertical extent of MHWs, leading to surface- and bottom-trapped events with shorter durations and intensities. Additionally, MHW initiation and termination across depths was consistently linked to anomalously low and high coastal upwelling, respectively. This study highlights the need for expansion of subsurface monitoring of MHWs globally amid a warming planet.

## Introduction

Rising ocean temperatures, driven by anthropogenic climate change, are causing more frequent and intense marine heatwaves (MHWs), which are prolonged periods of anomalously warm waters^1^. MHWs have caused long-term damage to marine ecosystems, threatened critical ocean-based economies, and are among the greatest threats to marine biodiversity on the planet^1–3^. MHWs have been linked with adverse ecological impacts across trophic levels; the loss of foundational species like seagrasses, kelps, and corals; mass mortality of ecologically and economically important species; and associated losses in ecosystem services^4^. MHWs can influence and co-occur with other stressors, leading to compound extreme events like low-pH, low dissolved oxygen, or harmful algal bloom (HAB) multi-stressor events^5–7^. For example, along the California Current, the 2014–2016 North Pacific Marine Heatwave contributed to the decimation of more than 90% of coastal kelp forests in some areas and the collapse of the $44 M Northern California abalone fishery^8^.

While satellite-based sea surface temperature (SST) measurements have yielded considerable insight into the spatiotemporal trends and dynamics of MHWs, they are limited to detecting surface MHWs and cannot delineate the subsurface structure or the presence of MHWs at depth^9^. Only recently have researchers begun to unravel these extremes “hidden” from satellites at depth, with the majority of subsurface and bottom MHW studies relying on hindcast reanalysis products (e.g., Global Ocean Reanalysis and Simulations—GLORYS)^10–14^. Reanalysis products are usually necessary to observe subsurface MHWs because of the lack of long-term continuous temperature series with the daily measurements necessary for MHW detection^15–17^. While bottom MHW studies are rare compared to surface MHW studies, significant findings include that subsurface MHWs can exist in the absence of surface MHWs and can persist longer, with a greater intensity, than surface events. Therefore, subsurface MHWs may have a greater cumulative thermal stress and ecosystem impact^10,18–24^. MHWs also exhibit considerable vertical variability—largely influenced by mixed layer dynamics—and can propagate vertically through the water column^19–22,24,25^.

While most studies depend on reanalysis products, others have investigated subsurface temperature anomalies during MHW events using infrequently sampling platforms such as monthly ship-based profiles or Argo floats^7,9,24–27^. Each of these approaches has their own inherent limitations associated with the scarcity of historical observations through both time and space^7,9,10,28^. For example, ocean reanalysis products are susceptible to errors in the evolution of subsurface temperatures in areas with limited historical observations^9,10,18^. As a result of sparse subsurface measurements, the characterization and prediction of subsurface MHWs remains a major global challenge.

MHW studies face additional challenges in shallow (< 20 m depth) nearshore coastal regions, which host some of the most productive, but also vulnerable, marine ecosystems^29^. This is true both for surface MHWs due to known biases and limitations of remotely sensed SST in the coastal zone, and for subsurface MHWs, due to the lack of data availability and the high resolution needed for numerical modeling. Satellite-based measurements in shallow coastal zones are susceptible to signal contamination from land run-off, aerosols, and water vapor —particularly in Eastern Boundary Current Upwelling Systems (EBUS) with seasonally persistent marine layer fog—as well as coarse spatial resolution that does not capture smaller coastline features^30–32^. For subsurface/bottom MHWs, there is a lack of long-term continuous data in the nearshore: Argo floats are only found in deeper open-ocean waters^24^, sustained ship-based profiling programs are typically monthly or quarterly^7^, established glider lines are limited to deeper shelf waters at an insufficient temporal resolution^33^, and most long-term nearshore monitoring stations and buoys are fixed at a single vertical point—usually the surface. Moreover, hindcast reanalysis products are limited by their grid cell size (typically 8 km or larger horizontally) and may not accurately capture key small-scale physical processes and important MHW drivers in the nearshore^11,24,34,35^. Even computationally expensive, high-resolution regional models may still have biases or limited ability to validate and assimilate long-term in-situ data^23,36^. Several studies have characterized subsurface temperature anomalies and MHWs using long-term moorings in deeper (> 50 m depth) shelf waters and found that seasonal stratification and local dynamics significantly impact the evolution of these events^17,22,37^. However, we are not aware of any studies that have examined the vertical structure of MHWs in the shallow (< 20 m depth) nearshore using in-situ data, despite the high productivity and ecological importance of nearshore regions.

**Fig. 1:**
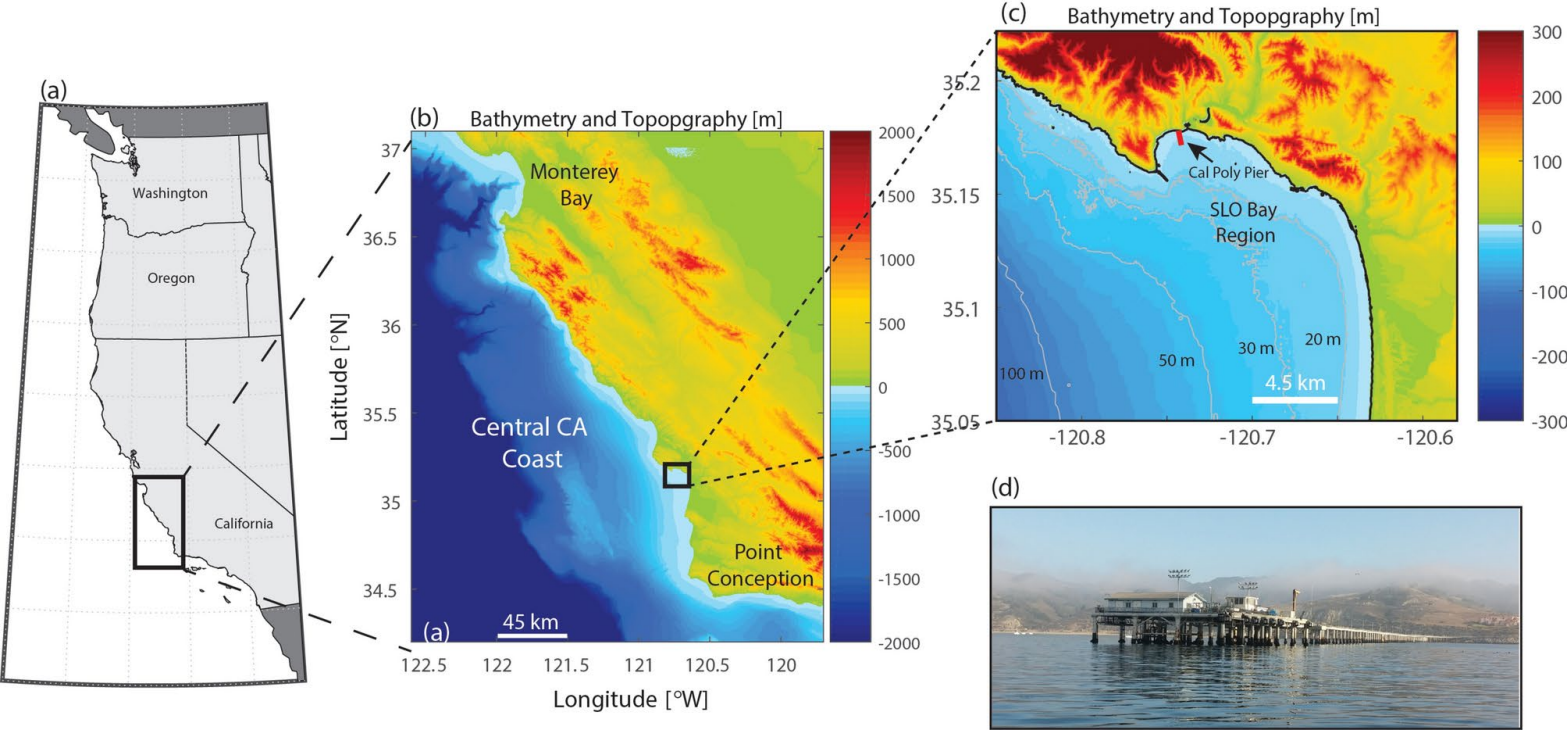
Study area showing the (**a**) United States West Coast and (**b**) bathymetry and topography of the Central California region. The inset in panel (**c**) shows the details of the SLO Bay region, as well as the location of the approximately 1 km long Cal Poly Pier (red line). (**d**) Photo of the Cal Poly pier looking to the northwest.

In this contribution, we quantify and characterize the vertical structure of MHWs at a shallow nearshore site located along the California Current EBUS. We take advantage of a unique long-term (2005–2023) dataset from an automated, high-resolution vertical profiling instrument located at the end of the California Polytechnic State University Pier at ~ 10 m depth (Fig. 1, site description in section “Site description”). This study provides the first known investigation into subsurface MHWs in a shallow (< 20 m depth) coastal region, offering an unprecedented view and critical insight into the vertical structure of nearshore MHWs and their potential drivers. The findings have important implications for predicting the impacts to and vulnerabilities of marine ecosystems in a warming climate.

## Results

### Climatology and General MHW Characteristics

**Fig. 2:**
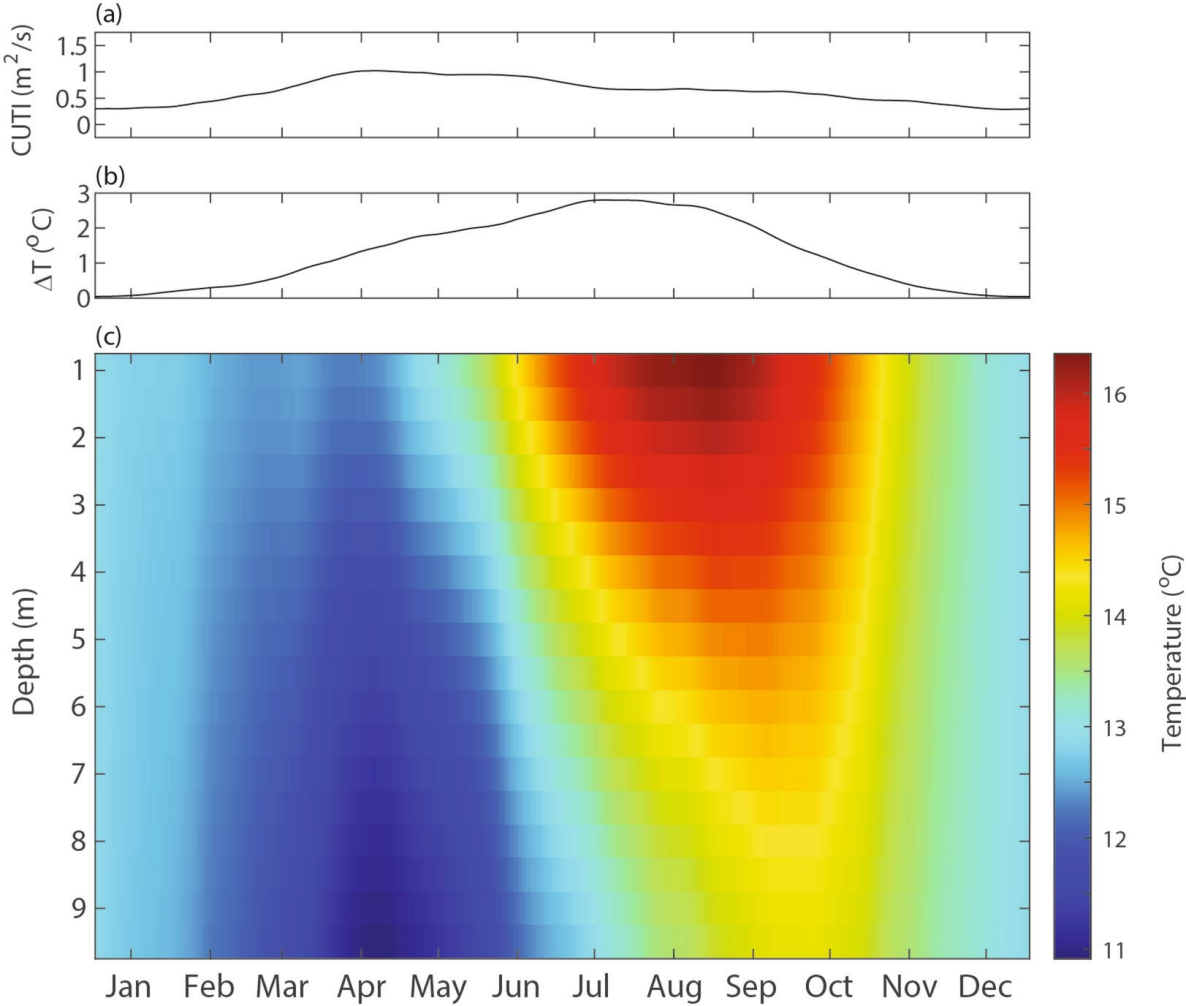
Annual climatology of the (**a**) Coastal Upwelling Transport Index (CUTI), (**b**) stratification index (average temperature difference between 1 and 9.5 m depths), and (**c**) temperature structure over depth (0.5 m bins) from the automated profiler at the end of the Cal Poly Pier.

The Coastal Upwelling Transport Index (CUTI, see section “Environmental data”) climatology showed expected seasonal variations with peak upwelling in April and May, moderate upwelling through the summer, and minimal upwelling during the winter months. The peak upwelling season aligned with periods of colder water, especially in the lower portion of the water column based on the temperature climatology (Fig. 2). The seasonal upwelling relaxation period (June through October) was characterized by a temperature stratified water column (> 2 °C between surface and bottom). During the late fall and winter months, the water column was typically well-mixed with minimal temperature stratification.

**Fig. 3:**
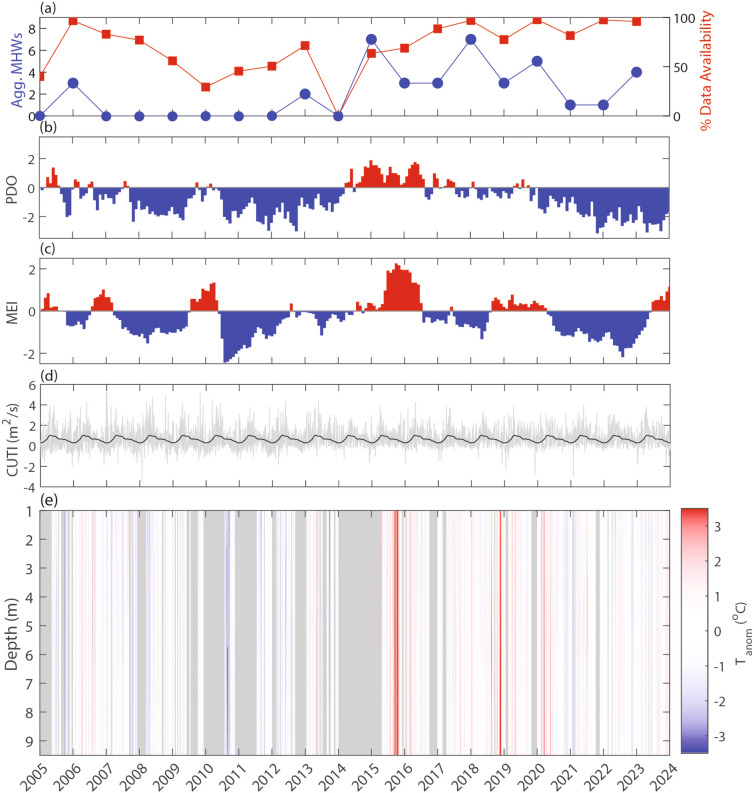
Time series of (**a**) annual aggregate MHW events (left, blue) and annual data availability (right, red), (**b**) monthly PDO index (red = positive/warm phase, blue = negative/cold phase), (**c**) monthly MEI index (red = positive, blue = negative), (**d**) Coastal Upwelling Transport Index (CUTI) climatology (black) and daily values (gray), and (**e**) temperature anomalies over depth (0.5 m bins) from the automated profiler at the end of the Cal Poly Pier. In panel (e), gray indicates missing data.

Across the entire time series, we detected 39 aggregate MHWs (see section “MHW characterization”), with a mean of 2.2 aggregate MHWs per year. However, there was large interannual variability in event frequency, with most events occurring between 2015 and 2021 and a notable absence of MHWs from 2007 to 2012 (Fig. 3). The interannual variability of aggregate MHWs aligned with variations in the basin-scale climate modes, with aggregate MHWs common during the positive phase of the Pacific Decadal Oscillation (PDO) and Multivariate El Niño Southern Oscillation Index (MEI). The 39 aggregate MHWs encompassed 522 individual MHWs across each depth bin. For individual MHWs averaged across all depths, the mean duration was 10.9 days with a standard deviation of 6.4 days and the mean intensity was 2.0 °C, with a standard deviation of 0.5 °C. Notably, the greatest MHW intensity was 4.9 °C in September 2015, with anomalies of at least 4.5 °C at all depths (depth fraction = 100% for this event).

### MHW depth and seasonal variability

**Fig. 4:**
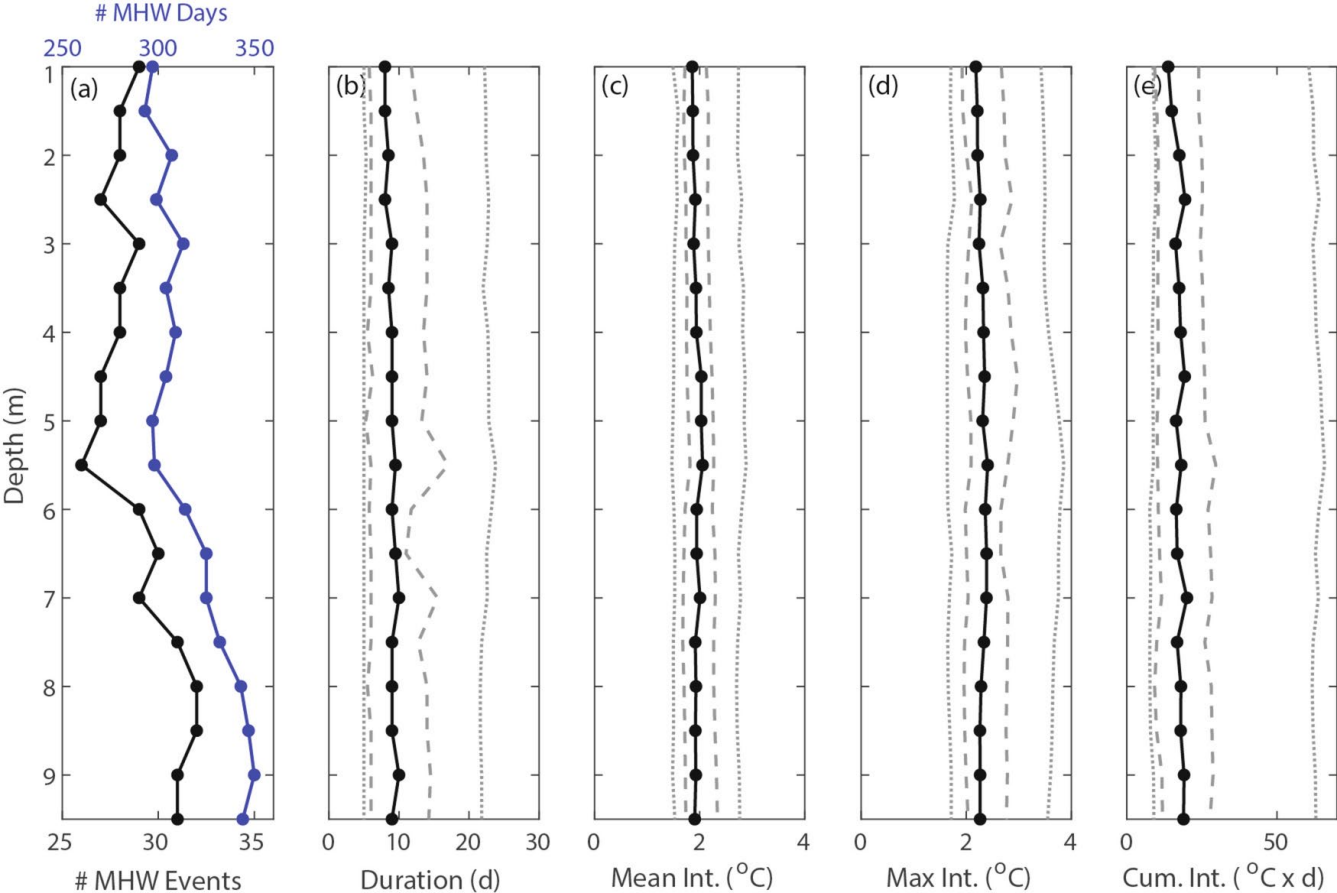
MHW statistics at various depths including the (**a**) number of MHWs (black, bottom axis) and total number of MHW days (blue, top axis), (**b**) MHW duration, (**c**) mean MHW intensity, (**d**) max intensity, and (**e**) cumulative intensity. Black lines denote the median value, dashed grey lines the 25th and 75th quartiles, and dotted grey lines the 10th and 90th percentiles. Percentiles are calculated across all MHW events at each depth.

Small variations in the number of MHW events and total number of MHW days were present across all depths, with smaller values at the surface (29 events, 297 MHW days) compared to the bottom (31 events, 344 MHW days) depth. There was minimal variability of MHW duration and intensity metrics with depth (Fig. 4). We note that these average characteristics across depths do not capture differences in event-scale variability (see section “event-scale variability and vertical co-occurrence”).

**Fig. 5:**
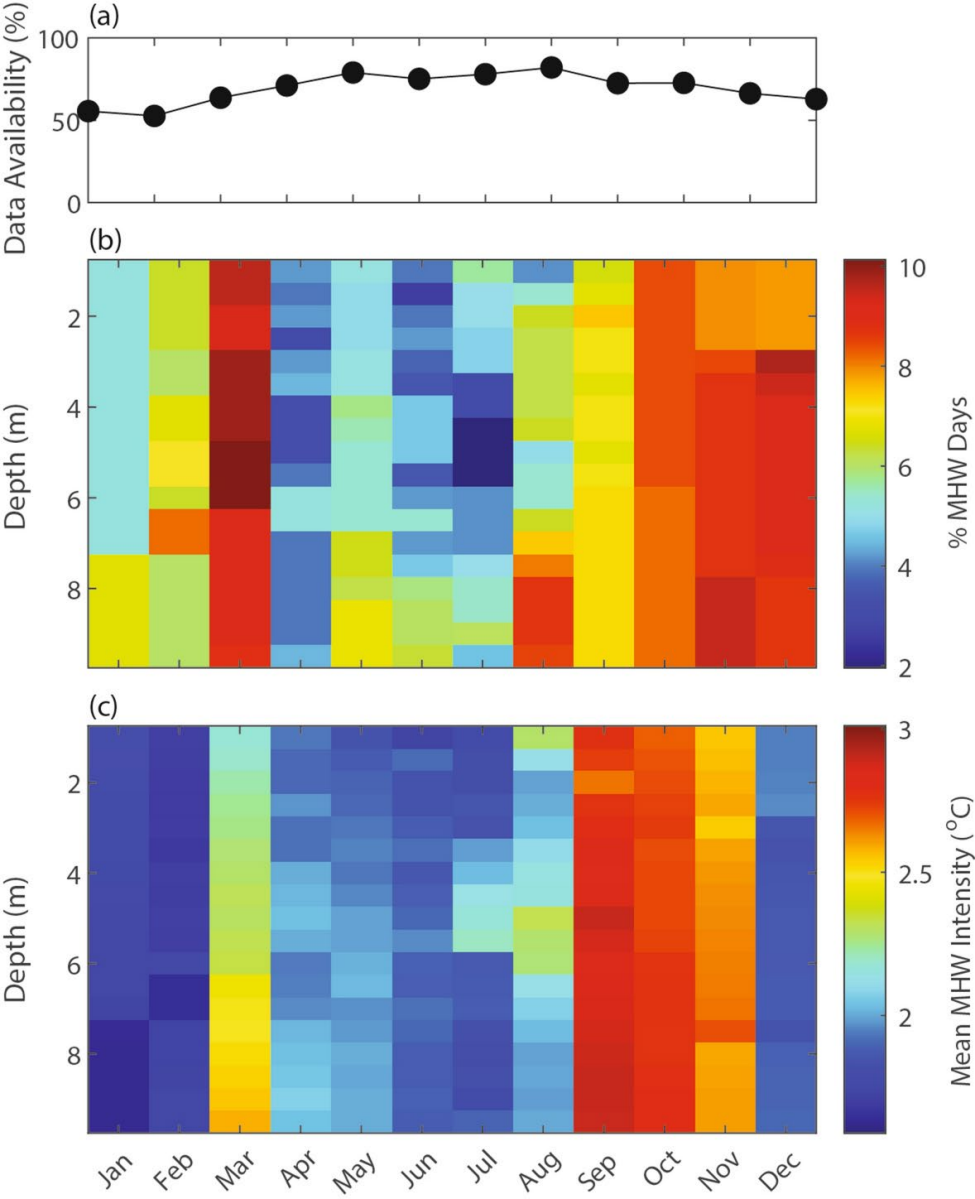
(**a**) Data availability, (**b**) percent occurrence of MHW days at each depth, and (**c**) mean MHW intensity at each depth, all by month. Percent occurrence of MHW days is normalized by data availability in that month. The mean MHW intensity is the average intensity of MHW days experienced in that month.

October, November, and December exhibited the greatest percentage of MHW days (Fig. 5b). The high number of MHW days in March was driven by two long events: a 32-day MHW in 2016 and a 26-day MHW in 2020. MHWs had the highest mean intensity in the late summer and early fall (September to November), with the highest intensity in September (Fig. 5c). September’s high intensity was partially driven by the most intense MHW in the record in 2015. The relative scarcity of MHW days from April to August aligned with the major upwelling season and strong seasonal stratification in the water column (Fig. 5b). Notably, August and September, two months with the strongest climatological stratification (Fig. 2), also displayed the most vertical variability in MHW occurrence (Fig. 5b).

### Event-scale variability and vertical co-occurrence

#### MHW event-scale vertical variability

The event-scale dynamics of MHWs revealed depth-dependent characteristics not present in the long-term averages. There were seven surface-trapped events that only occurred in the upper half of the water column and eight bottom-trapped events that only occurred in the lower half of the water column (see Supplementary Figs. S1 and S2 for examples). Only three of 39 aggregate MHW events had a 100% depth fraction (see Fig. S3 for an example), and roughly half (20 out of 39) of aggregate MHW events occurred with a depth fraction less than 75%. Surface- and bottom-trapped events, as well as other MHWs with low depth fractions, were more likely to occur in the stratified summer period, while the MHWs that occupied a large portion of the water column with high depth fractions were observed during periods of weak stratification in the late fall and winter (more details in section “local oceanic conditions at the event scale”). Event initiation and termination also commonly coincided with negative and positive CUTI anomalies (CUTIa), respectively, for both high- and low-stratification events (Supplementary Figs. S1 S2, and S3; see also more details in section “Local oceanic Conditions at the event scale”).

#### Vertical co-occurrence

**Fig. 6:**
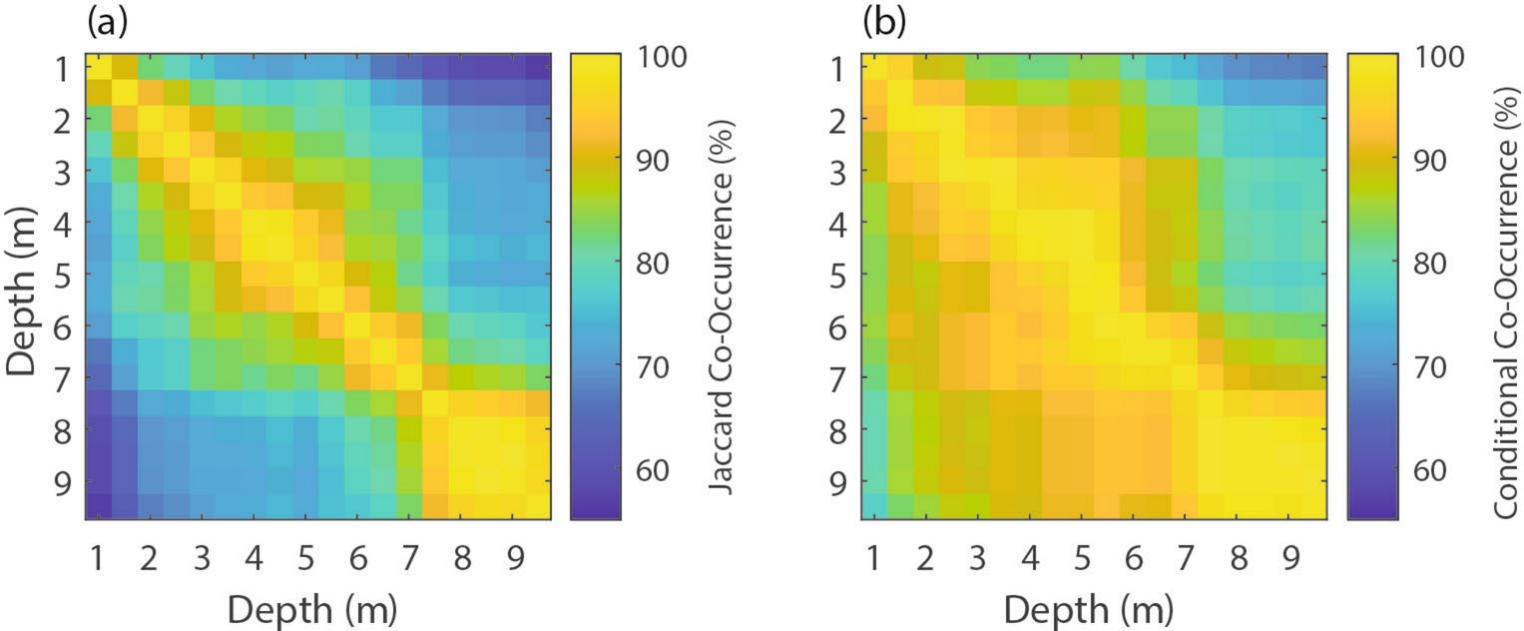
Co-occurrence matrices of MHW events between different depths defined with (**a**) the Jaccard index (Eq. 1) and (**b**) the conditional co-occurrence (Eq. 2). For conditional co-occurrence, each value corresponds to the probability that the row depth is in an active MHW given that a column depth is in an active MHW (e.g., top right corner would be the percent co-occurrence of the surface being in a MHW given that the bottom is in a MHW).

Examination of the Jaccard Index co-occurrence matrix (Eq. 1, Fig. 6a) and conditional co-occurrence matrix (Eq. 2, Fig. 6b) reveals strong vertical variability in MHW co-occurrence with distinct regimes. As expected, the highest co-occurrence was found adjacent to the diagonal (100% along the diagonal by definition) of both matrices since closer depths are more likely to experience MHWs at the same time. There was also notably higher co-occurrence in the bottom third of the water column—these deeper depths tended to act as a contiguous block more than the shallower depths (see yellow square in bottom right corners of Fig. 6a and b). This block also indicates that bottom-trapped events exhibited a higher depth fraction than surface-trapped events.

The lower rates of co-occurrence away from the diagonal indicate that MHWs did not occur at the same time across all depths, for example, during surface- and bottom-trapped events. The Jaccard index varied down to ~ 50% in the corners of Fig. 6a, which means that MHW days at the surface and bottom only co-occurred around half of the time. The surface (depth = 1 m) had the smallest Jaccard index with other depths, especially when comparing the surface to the bottom of the water column. Furthermore, based on conditional co-occurrences, if there was a MHW day at the top surface bin, there was a ~ 75–85% co-occurrence with a MHW day at depth (Fig. 6b). However, when there was a MHW day at depth, there was only a ~ 60–70% co-occurrence with a MHW day at the surface (Fig. 6b). This is an important finding since it means that approximately one third of bottom MHW days exist independently of, or do not occur with, a surface MHW day. Indeed, 11 out of 39 aggregate MHW events never appeared at the surface.

Aggregate MHW events where co-occurrence at different depths took place displayed an initiation time lag between depths. The average time lag across all MHWs and all depth pairings was 0.34 days, with the average time lag between pairs of depths never exceeding 1 day (Supplementary Fig. S4). However, depth pairings in the upper portion of the water column showed lower time lag variability (standard deviation less than 1.5 days, see blue square in Supplementary Fig. S4b), whereas the depth pairings in the bottom portion had higher variability (e.g., 90th percentile lag between 5 and 9 m was 3 days). Aggregate MHWs had a slight tendency to start lower in the water column and propagate upwards (Supplementary Fig. S4a), but that tendency was a relatively weak signal with high variability.

### Local oceanic conditions at the event scale

#### Upwelling anomalies and event initiation/termination

**Fig. 7:**
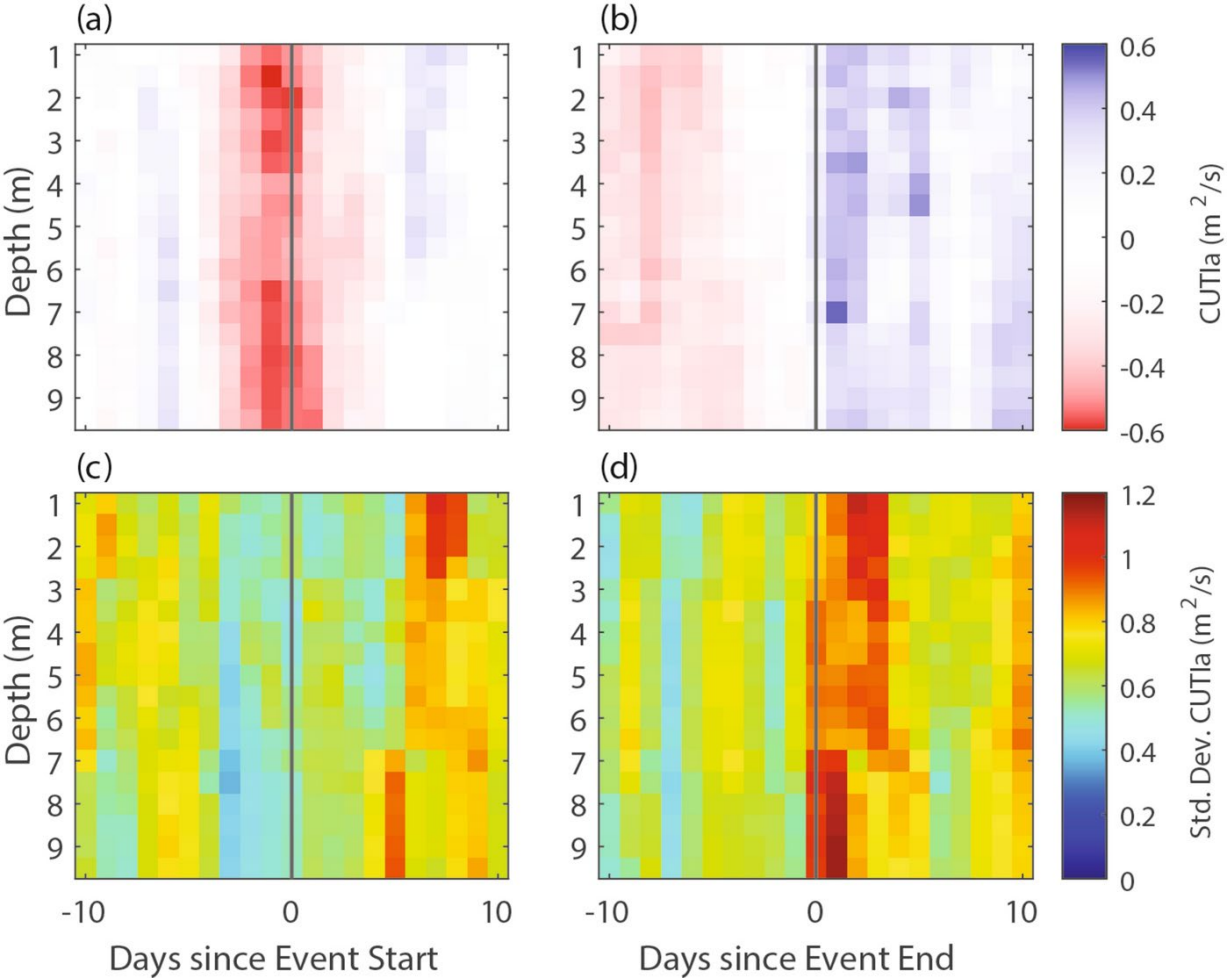
Composite averages of the CUTI anomaly before and after the (**a**) initiation and (**b**) termination of MHW events. Standard deviations are shown in panels (**c**) and (**d**).

To further investigate the relationship between MHW initiation/termination and upwelling anomalies, we calculated composite averages of the CUTIa across all MHWs and all depths. Negative CUTIa consistently coincided with MHW initiation (Fig. 7a). In fact, all but one aggregate MHW had a negative CUTIa within two days before event initiation. A negative CUTIa value indicates either anomalously low upwelling if CUTI is positive, or downwelling if CUTI is negative. Approximately 59.0% (23 out of 39) of the aggregate MHW events experienced downwelling (negative CUTIa and CUTI) within two days before event initiation. For context, downwelling occurred on less than 20% of days in the full time series.

The transition to anomalously strong upwelling (positive CUTIa) consistently coincided with MHW termination (Fig. 7b). However, CUTIa at termination was more variable (Fig. 7d) than at the initiation (Fig. 7c). Furthermore, 29 of 39 aggregate MHW events had a positive CUTIa within one day on either side of event termination, suggesting that there are additional important drivers of MHW termination (see “Discussion and conclusions”). For reference, the average CUTIa at event initiation and termination, respectively, was approximately equal to the interquartile range (IQR), or the 25th and 75th percentiles, of CUTIa values across the entire time series (– 0.5 to 0.5 m^2^/s).

#### Depth fraction and stratification

**Fig. 8:**
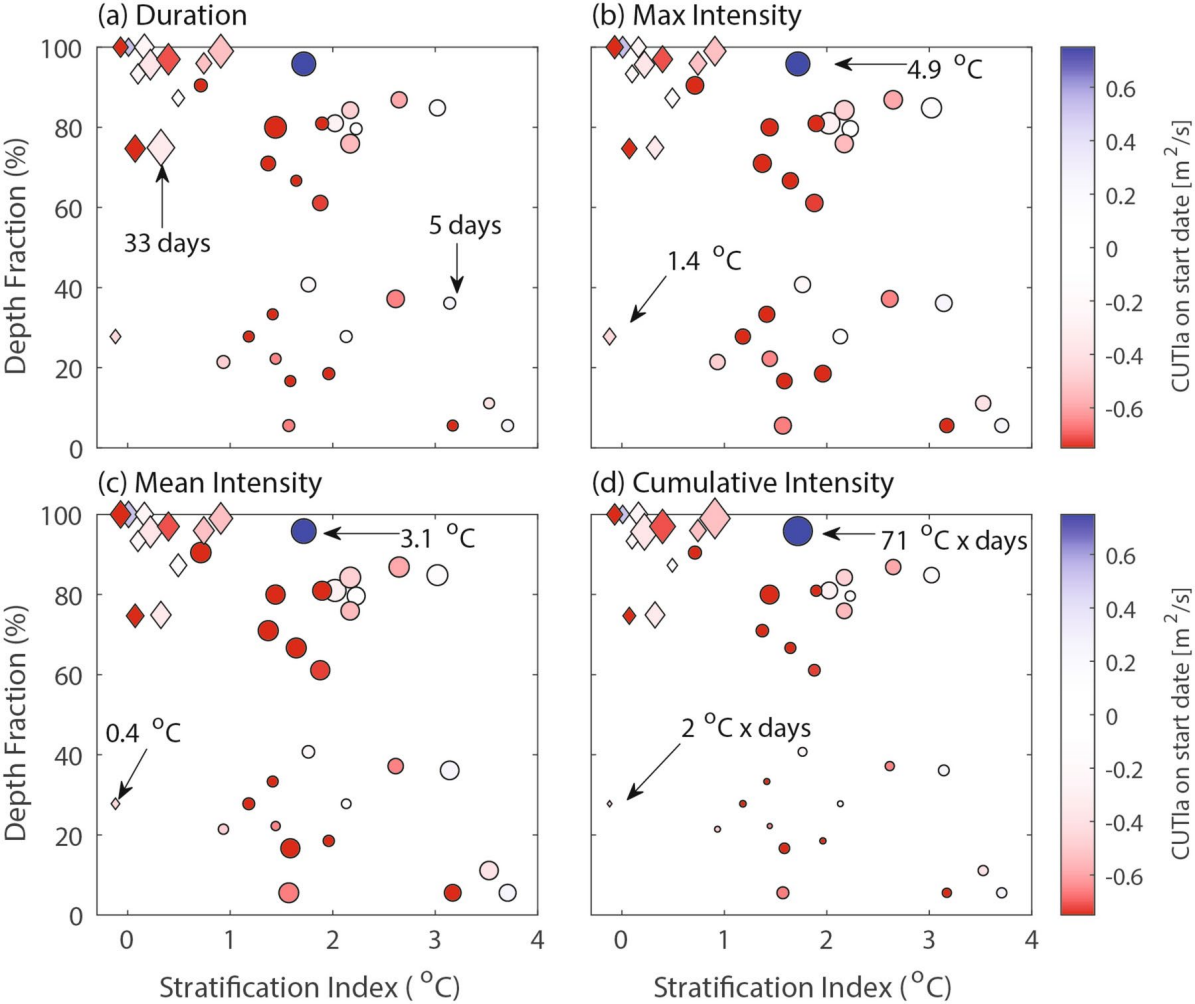
Scatter plots of MHW depth fraction as a function of the water column temperature stratification for all aggregate events (n = 39). Aggregate MHW events are scaled by (**a**) duration, (**b**) average max intensity across active depths, (**c**) average mean intensity across active depths, and (**d**) average cumulative intensity across active depths. The events are colored by the CUTIa at event initiation. Diamond markers denote MHWs that start during the non-upwelling season (October to March), while circles denote the upwelling season (April to September).

To further explore vertical variability, we examined how water column stratification impacts the depth fraction of aggregate MHW events. A distinct clustering appears in the relationship between depth fraction and stratification index (see section “MHW characterization”). During the winter months, aggregate MHWs consistently displayed a higher depth fraction, and this regularly occurred during periods of low stratification (Fig. 8, diamonds). These high depth-fraction aggregate MHW events also displayed a higher mean duration (Fig. 8a), intensity (Fig. 8c), and cumulative intensity (Fig. 8d), thereby imparting the greatest water-column integrated thermal stress. On the other end, the highest stratification events coincided with the lowest depth fractions, as well as some of the smallest durations and intensities (Fig. 8). For moderate stratifications, depth fractions were highly variable. Across all aggregate MHW events, the CUTIa was consistently negative at event initiation (colorbar in Fig. 8), but there was no clear relationship between the magnitude of the CUTIa and the depth fraction or stratification index. Finally, the max intensity was roughly the same across all aggregate MHW events (Fig. 8b).

## Discussion and conclusions

This study is the first known analysis of subsurface MHW structure in a shallow nearshore upwelling system. While previous studies of subsurface and bottom MHWs have relied on relatively data-limited reanalysis models and sparse in situ sampling, we utilized a unique and novel temperature series spanning nearly two decades with continuous vertical coverage from the surface to the sea floor in a shallow coastal location in central CA^10,11,13^. Importantly, we found that ~ 1/3 of bottom MHW days are hidden at depth and do not co-occur with surface MHW days (Fig. 6b). Despite being in a shallow depth of only 10 m, surface and subsurface extreme temperature anomalies (MHWs) were disconnected and occurred independently from one another, which has major implications for the monitoring and predicting of MHWs in ecologically and economically important nearshore systems. This work also corroborates findings from other studies on bottom MHWs that show subsurface dynamics and MHWs can be distinct from their surface counterparts, implying that satellite detection is not a reliable method of detecting and/or quantifying subsurface MHWs even in shallow nearshore environments^10,21,25^. This underscores the importance of sustained in-situ monitoring programs that provide depth-resolved and/or bottom measurements in a variety of systems.

Furthermore, while we found total MHW days varied slightly with depth, with bottom MHWs occurring on ~ 17% more days compared to the surface, average MHW metrics (duration and intensity metrics) did not vary significantly with depth (Fig. 4). This finding contrasts with studies that found deeper bottom MHWs lasting longer and with greater intensities compared to surface MHWs^19–21,25,34^. However, the referenced studies measured MHWs in much deeper shelf waters and/or the open ocean. The difference in behavior of subsurface MHWs may stem from the unique dynamics found in shallow nearshore systems, which are influenced by higher-frequency processes that impact both surface and bottom boundary layer dynamics^38^. For example, in this study, we found that wind-driven upwelling anomalies were a primary driver of MHWs at all depths, with 97% of aggregate event initiations coinciding with negative CUTIa (anomalously weak upwelling or downwelling) and 74% of aggregate event terminations with positive CUTIa (anomalously strong upwelling) (Fig. 7a, b). Previous work from this region documented that upwelling-relaxation cycles with a one-to-two-week period resulted in large advective fluxes of cold (upwelling) and warm (relaxation) water into the upwelling bay system, with highly variable stratification and mixed layer dynamics^39,40^. Given the typical timescale of these upwelling-relaxation cycles, as well as the strong connection of MHW initiation/termination to upwelling anomalies, the upwelling-relaxation cycles at least partially control the duration of MHW events in this system. While other studies have found that upwelling can influence MHW occurrence^17,22,29^, this study demonstrates coastal upwelling as a primary driver of both surface and subsurface MHWs, a finding that is likely relevant to coastal sites in EBUS globally. Future work should investigate the connection between subsurface MHWs in nearshore upwelling systems and adjacent shelf waters, especially since the offshore stratification, thermocline depth, and topographic slope interact to influence the depth of origin, and temperature, of cross-shelf upwelling-driven flows (i.e., the slope Burger number)^41,42^. We stress the need for high-resolution numerical models of shallow nearshore systems to investigate detailed dynamics of MHW events, but these models should be validated by in-situ depth resolved measurements over sufficiently long timescales.

In addition to upwelling anomalies, we also found that stratification was a key control on MHW characteristics. During fall and winter months when stratification was minimal, MHWs occurred over a much higher depth fraction with a larger cumulative thermal stress (Fig. 8). The greatest number of MHW days also occurred during the fall and winter months, with the clear seasonality likely impacting the complex life cycles of some marine species^4^. During summer months, strong stratification limited depth fraction and resulted in a greater number of surface- or bottom-trapped events, with important ecological considerations and potential impacts. Seasonal variation of MHW behavior likely occurs because stratification is an important control on vertical diffusion of heat, mixing, and depth penetration of temperature anomalies^7^. Future work, potentially with high-resolution numerical models, should investigate the relative role of advective fluxes, vertical transport and mixing, and air-sea heat fluxes in controlling nearshore MHWs across different seasons and forcing conditions. For example, we found that during downwelling or anomalously low upwelling events (negative CUTIa) in the stratified summer period, the transport of warm water anomalies can lead to bottom-trapped events that are limited to the benthic environment. Moreover, previous studies have shown that MHW duration and cumulative intensity positively correlate with MHW horizontal spatial extent^9,20,43^ and since winter events in this study tended to be longer duration with a higher cumulative intensity, it is also possible that they have a much greater horizontal spatial extent and overall ecosystem impact. On the other hand, it is possible that summer MHW events are localized to the semi-enclosed embayment, since an upwelling shadow system develops inside the bay during the summer upwelling season, and high retention times can lead to increased stratification and warming of the bay waters relative to outside the bay^39,40,44^. High retention times within the bay are also favorable for near-bottom hypoxia^45^. Bottom-trapped MHWs in this system, and upwelling shadows in general, may therefore coincide with multiple stressor compound events (e.g. MHWs coinciding with low dissolved oxygen, low pH, and/or HAB events) in a way that surface MHWs do not.

The number of MHWs annually at this site largely mirrored the general regional results from an adjacent coastal location^29^, with fewer MHWs during cool phases of the PDO prior to 2014. This was followed by increases in MHW events later in the record with the largest number of aggregate MHWs documented in 2015 during the record El Niño [see also peak in Dalsin et al. (2023)^29^] and Northeast Pacific Marine Heatwave from 2014–2016 that spanned the US West Coast (note that the profiler in this study had no data in 2014 during the so-called “warm blob” event—see Fig. 3)^46^. This linkage suggests a partial regional coherence in MHW events, but investigating the detailed dynamical links between larger-scale oceanographic conditions and nearshore MHWs, and in particular the vertical structure of these events, should be addressed in future work. Understanding this connection remains a grand challenge due to, for example, a lack of long-term subsurface data, satellite biases in coastal regions, and nearshore modeling challenges at a high enough resolution over long enough time periods (see section “Introduction”).

Reliable predictions and forecasting of subsurface and bottom MHWs, which are currently limited, will require more sustained monitoring programs that can characterize subsurface MHWs hidden at depth, particularly in coastal regions^9^. These measurements can be used to develop the robust and spatially resolved temperature climatologies needed to accurately quantify subsurface MHWs, but they can also improve validation of high-resolution numerical models and reanalysis products for subsurface MHW characterization^9–11,13^. With more accurate forecasts, stakeholders and resource managers can adapt active management strategies and proactive approaches that mitigate potential impacts during high-risk periods like strong El Niño years^47^. These results suggest that long-term forecasts of large-scale basin-scale climate modes (PDO, MEI) in the North Pacific, in conjunction with short-term weather forecasts of regional wind-forcing (upwelling), could be used to develop a MHW risk metric, and will be considered in future work. Moreover, reliable prediction and forecast systems will be sensitive to future climate-change induced changes to stratification, wind-driven upwelling, and the frequency and intensity of basin-scale climate modes^48^.

This study documented, for the first time, some of the unique characteristics of subsurface MHWs in a shallow nearshore upwelling system. Expansion of subsurface monitoring and additional studies of subsurface MHWs in EBUS are needed globally since EBUS may serve as thermal refugia for cold water organisms in a warming planet and provide reprieve from globally increasing MHW trends^49^.

## Methods

### Site description

Temperature measurements were collected in San Luis Obispo (SLO) Bay, a small coastal embayment located in central California in the North Pacific Ocean (Fig. 1). Like the larger California Current EBUS, the SLO Bay region hosts giant kelp forests and valuable commercial and recreational fisheries^50,51^. The habitats adjacent to SLO Bay also support high-value fisheries, including groundfish and Dungeness Crab, and as such, SLO Bay is home to a major regional fishing port^50,51^. SLO Bay also lies adjacent to the Pt. Buchon marine protected area. In this region, seasonally variable coastal upwelling is the dominant driver of physical, chemical, and biological variability; upwelling also drives the flux of deep, cold, and nutrient-rich waters into nearshore regions fueling primary productivity^44,45,52,53^. The major upwelling season typically lasts from March to September, with peak upwelling occurring in the spring and moderate upwelling in the later summer months, the latter of which coincides with significant water column stratification^44^. In the weak upwelling season (late fall and winter months), the water column is typically well-mixed^44^. Overall, circulation patterns and temperature variability in SLO Bay are controlled by upwelling seasonality, although higher-frequency upwelling-relaxation cycles (1–2-week period) and strong local diurnal wind forcing can also significantly influence dynamics^39,40,44^. The timing of seasonal warming and cooling is consistent across nearshore sites in the region, but climatological surface temperatures within the bay are up to ~ 1 °C warmer than a nearby location outside of the bay where previous work on nearshore MHW variability has focused^29,44^. SLO Bay also has a high propensity for significant hypoxic events and HABs, and thus it is important to understand MHW drivers to aid future studies on the potential for multi-stressor events with synergistic, adverse impacts^45,52,53^.

### Temperature measurements

Temperature measurements throughout the water column in a depth of approximately 10 m were collected using a winch-controlled autonomous profiler at the end of the ~ 1 km long California Polytechnic State University Pier in SLO Bay. The automated profiling system contains several sensors, including a Sea-Bird electronics conductivity-temperature-depth (CTD) sensor^44^, and is supported by the Central and Northern California Ocean Observing System [CeNCOOS; (https://data.caloos.org/?&sensor_version=v2#metadata/103545/station/data)]. Temperature measurements were collected starting in May 2005 and are analyzed here through the end of 2023, resulting in a nearly 19-year dataset. Approximately every 30 min, the profiler lowers to a water depth of 1 m and equilibrates for one minute. Then, the profiler descends at ~ 4 cm/s to the sea floor. Any measurement more than five standard deviations from the median temperature of each profile was removed, and temperatures were binned into 0.5 m depth increments. Daily averages (see section “MHW detection”) were calculated at each depth bin, resulting in a 19-year daily-averaged temperature time series at each 0.5 m depth increment from 1 to 9.5 m (18 depth bins total). One day gaps were linearly interpolated, and only days with temperature measurements at all depths were considered. After interpolation and intersection, 70% of days had temperature values (see Figs. 3 and 5 for data availability).

### MHW detection

MHWs were detected according to the Hobday et al.^54^ definition as “contiguous periods of anomalously warm water,” with a minimum of five consecutive days exceeding the 90th percentile temperature threshold for a given day of the year^54^. MHWs separated by two days or less were combined into a single MHW. While the 19-year data timeframe is less than the optimal length of 30 years for calculating a climatology, the shortened time series likely does not have a significant impact on MHW characteristics or major conclusions^55^ [see Schlegel et al. (2019) for a detailed discussion of suboptimal datasets and associated uncertainties in MHW detection]. MHWs were detected independently for each depth bin. Following Hobday et al.^54^, the seasonal climatology for each depth was calculated by averaging data from all years that were within an 11-day window centered on a given day of the year; the same data were used to estimate the 90th percentile threshold^54^. No detrending of the temperature time series was done following Gupta^56^. MHW events were detected and characterized using a MATLAB MHW Toolbox^57^. The intensity of a MHW is defined as the daily-averaged temperature minus the climatological average for that day of the year. The cumulative intensity of an individual event is defined as the daily-averaged temperature minus the climatological average integrated over the MHW duration. The maximum intensity of a MHW is the greatest difference between the daily-averaged temperature and climatological average during an individual MHW.

### MHW characterization

We calculated the vertical co-occurrence of MHWs between each depth with two methods: the Jaccard index and the conditional co-occurrence. First, the Jaccard index is defined as the intersection of MHW occurrence at two depths divided by the union of MHW occurrence at those depths^58^. That is, for a set of MHW days at depth *a* and depth* b* (*MHW*_*a*_ and *MHW*_*b*_, respectively), the Jaccard index is:

1 $$J_{a,b} = J_{b,a} = \frac{{\left| {MHW_{a} \cap MHW_{b} } \right|}}{{\left| {MHW_{a} \cup MHW_{b} } \right|}} \times 100,$$

where the multiplication by 100 converts from a fraction to percentage. Thus, the Jaccard index measures the similarity of MHW occurrences between two depths relative to all MHW days across those depths, where *J(a,b)* = 0% signifies that depths *a* and *b* never experience a co-occurring MHW and *J(a,b)* = 100% equates to depths *a* and *b* having 100% overlap of all MHW days at those depths.

We termed the second method we used to quantify co-occurrence the “conditional co-occurrence”, which is the intersection of MHW occurrence at two depths divided by MHW occurrence at a single depth. That is, for a depth *a,* the conditional co-occurrence with depth *b* ($${C}_{a,b})$$ is the intersection of MHW days at *a* and *b* divided by the set *MHW*_*b*_,

2 $$C_{a,b} = \frac{{\left| {MHW_{a} \cap MHW_{b} } \right|}}{{\left| {MHW_{b} } \right|}} \times 100,$$

where the multiplication by 100 converts from a fraction to percentage. This calculation, analogous to a conditional probability, quantifies the percentage of MHW events that happen at depth *a* given that there is a MHW event at depth *b*. While the Jaccard index will produce a symmetric matrix when comparing different depths (*J(a,b)* = *J(b,a)*], the conditional co-occurrence yields an asymmetric matrix because MHW frequency and duration varies by depth. In Eq. (2), $$C_{a,b}$$ uses the denominator of *MHW*_*b*_, whereas $$C_{b,a}$$ uses the denominator of *MHW*_*a*_, both with the same numerator. As a trivial example, if depth *a* has a MHW on days 1, 2, and 3, and depth *b* has an MHW on day 1 but not days 2 or 3, the conditional co-occurrence of depth *a* given depth *b* ($$C_{a,b} )$$ is 100% (i.e., 100% of MHW days at depth *b* co-occur with MHW days at depth *a*), while the conditional co-occurrence of depth *b* given depth *a* ($$C_{b,a}$$) is 33.33% (i.e., only 33.33% of the MHW days at depth *a* co-occur with MHW days at depth *b*). The conditional co-occurrence was useful to identify whether there are surface MHWs not seen at the bottom, and vice versa.

We also investigated what we term “aggregate MHW events”, where an aggregate event begins when a MHW starts at any depth and ends when there is no longer an active MHW at any depth. During aggregate MHW events, we quantified the depth fraction (percentage of depths where a MHW was detected during an aggregate event, regardless of duration) and the bulk stratification index (average temperature difference between 1 and 9.5 m depth). For aggregate MHW events, intensity metrics are defined as the average intensity across depths that are in active MHW status during the aggregate event (e.g., the maximum intensity of an aggregate MHW active at 1 m, 1.5 m, and 2 m is the average of the maximum intensity of the MHW at those respective depths during the aggregate MHW). We also quantified the time lag in MHW initiation between the co-occurring depths of aggregate MHW events. Finally, we classified surface-trapped MHWs as those that occur only in the upper half of the water column (between 1–5 m), while bottom-trapped events occur only in the lower half of the water column (between 5.5–9.5 m).

### Environmental data

To assess the role of basin-scale climate modes on MHW interannual variability, we examined the MEI and the PDO^29^. The MEI measures the strength of El Niño (positive) and La Niña (negative) events and is defined using the leading combined empirical orthogonal function of SST, sea level pressure, zonal surface wind, meridional surface wind, and ocean-emitted radiation in the Pacific Ocean basin (https://psl.noaa.gov/enso/mei/). The Pacific Decadal Oscillation is an interdecadal climatic pattern of SST anomalies in the North Pacific calculated as the first mode of the empirical orthogonal function of SST anomalies in this region (https://www.ncei.noaa.gov/access/monitoring/pdo/).

To investigate the influence of coastal upwelling on MHW variability, we utilized the CUTI, which measures the vertical transport of water into the surface mixed layer^29,59^. CUTI is calculated daily at 1° latitudinal bins (35° N used here) over an area that extends 75 km offshore and is available since 1988 (see area in Fig. 2 of Jacox et al.^59^; https://mjacox.com/upwelling-indices/). We also calculated CUTIa, defined as the difference between the daily CUTI and the climatological CUTI value, with CUTI climatology calculated in the same way as temperature climatology (see section “MHW detection”) over the entire period of availability since 1988. We examined the composite average of CUTIa values at each depth across all MHW events to assess whether event initiation and/or termination were influenced by anomalous upwelling.

## Supplementary Information

Supplementary Information.

## Data Availability

The temperature data used here will be made available by the corresponding author upon reasonable request. All other data are publicly available.
