# Supplementary material for: Vertical structure of subsurface marine heatwaves in a shallow nearshore upwelling system
Source: Sci Rep. 2025 Feb 21;15:6353. doi: 10.1038/s41598-025-90565-4 (PMC11845478; doi:10.1038/s41598-025-90565-4)
Supplement: Supplementary file 1 — Supplementary Information. [file 41598_2025_90565_MOESM1_ESM.docx]

**Vertical structure of subsurface marine heatwaves in a shallow nearshore upwelling system**

Gavin Plume^1^, Ryan K. Walter^1*^, Isabelle Cobb^1^, Michael Dalsin^1^, Piero L.F. Mazzini^2^, Nathan P. Shunk^2^, Ian Robbins^1^, and Thomas P. Connolly^3^

^1^Physics Department, California Polytechnic State University, San Luis Obispo, CA, USA

^2^Virigina Institute of Marine Science, William and Mary, Gloucester Point, VA, USA

^3^Moss Landing Marine Laboratories, San José State University, Moss Landing, CA, USA

*Corresponding author ([rkwalter@calpoly.edu](mailto:rkwalter@calpoly.edu))

**Supplemental Information**


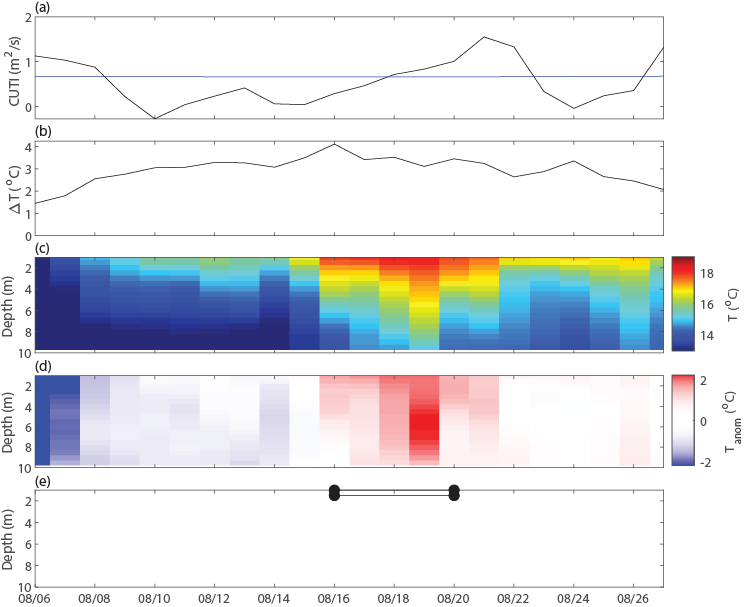


**Figure S1:** Event-scale characteristics of a surface-trapped aggregate MHW event in August 2020. (a) CUTI climatology (blue) and daily CUTI values (black), (b) stratification index (temperature difference between the 1 and 9.5 m depths), (c) temperature at various depths (0.5 m bins), (d) temperature anomalies (daily temperature minus climatology) at various depths (0.5 bins). Panel (e) highlights the MHW event initiation and termination (dots) over the duration of the MHW event (connecting line) at the depths of occurrence. Note the difference in temperature colorbar in panel (c) compared to Figures S2 and S3.


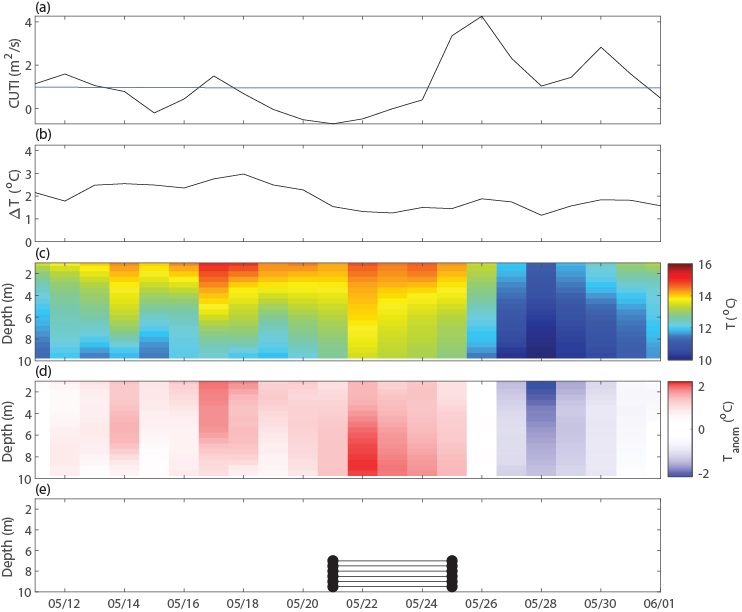


**Figure S2:** Event-scale characteristics of a bottom-trapped aggregate MHW event in May 2015. (a) CUTI climatology (blue) and daily CUTI values (black), (b) stratification index (temperature difference between the 1 and 9.5 m depths), (c) temperature at various depths (0.5 m bins), (d) temperature anomalies (daily temperature minus climatology) at various depths (0.5 bins). Panel (e) highlights the MHW event initiation and termination (dots) over the duration of the MHW event (connecting line) at the depths of occurrence. Note the difference in temperature colorbar in panel (c) compared to Figures S1 and S3.


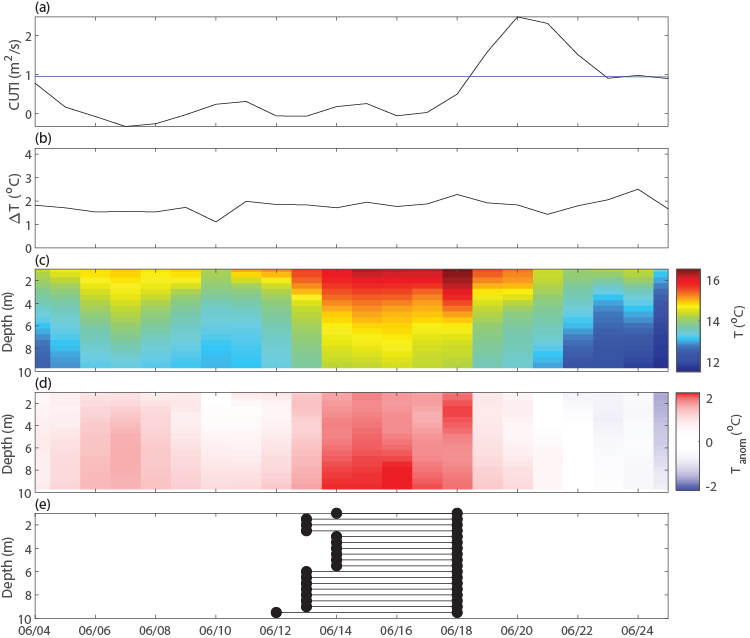


**Figure S3:** Event-scale characteristics of a full-water-column (depth fraction = 100%) aggregate MHW event in June 2023. (a) CUTI climatology (blue) and daily CUTI values (black), (b) stratification index (temperature difference between the 1 and 9.5 m depths), (c) temperature at various depths (0.5 m bins), (d) temperature anomalies (daily temperature minus climatology) at various depths (0.5 bins). Panel (e) highlights the MHW event initiation and termination (dots) over the duration of the MHW event (connecting line) at the depths of occurrence. Note the difference in temperature colorbar in panel (c) compared to Figures S1 and S2.


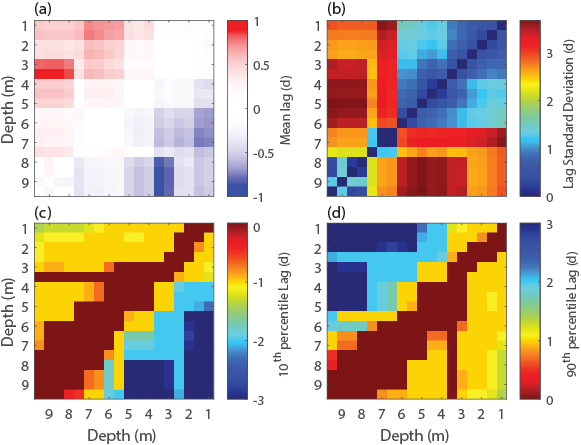
**Figure S4:** Co-occurrence lag matrices for MHW events. (a) Average lag between MHW start dates at two depths. (b) Standard deviation of lag for each depth pairing. (c) 10^th^ and (d) 90^th^ percentile lag for each depth pairing. For all values, the lag compares the row depth to the column depth [e.g., in panel (a), a positive lag indicates that the MHW event at the row depth lags the MHW depth at the column depth].
